# Topical Paromomycin and Gentamicin for New World Cutaneous Leishmaniasis in Panama

**DOI:** 10.4269/ajtmh.14-0040a

**Published:** 2014-06-04

**Authors:** Begoña Monge-Maillo, Rogelio López-Vélez

**Affiliations:** Tropical Medicine & Clinical Parasitology Infectious Diseases Department Ramón y Cajal Hospital Madrid, Spain E-mails: begomongem@gmail.com and rogelio.lopezvelez@salud.madrid.org

Dear Sir:

We read with interest the clinical trial reported by Sosa and others^1^ in which topical paromomycin/WR 279,369/gentamicin was compared with paromomycin alone for the treatment of New World cutaneous leishmaniasis (NWCL) caused by *Leishmania panamensis*. The authors concluded that the combination product may provide greater clinical benefit than paromomycin alone.

The authors stated that paromomycin plus methylbenzethonium chloride (MBCL) ointment has not been evaluated alone against *L. panamensis*. However, Krause and others^2^ published a non-randomized study of patients in Ecuador with *L. panamensis* NWCL, with paromomycin sulphate plus MBCL ointment administrated twice a day for 10 days or once a day for 20 days, compared with untreated patients. Cure rates were 85%, 85%, and 9%, respectively. Moreover Armijos and others^3^ in 2004 performed a randomized controlled trial in Ecuador in patients with *Leishmania guyanensis*, *Leishmania braziliensis*, and *Leishmania panamensis* NWCL, in which topical paromomycin plus MBCL ointment was compared with topical paromomycin sulphate plus urea, both twice a day for 30 days, compared with meglumine antimoniate for 10 days. The cure rates at 3 months were 79%, 70%, 92%, respectively.

The cure rates in these two studies were similar to those seen by Sosa and others^1^ with combination therapy (86%). Thus, good cure rates for *L. panamensis* NWCL can be obtained when paromomycin ointment is combined with other agents. However, this was not shown for *Leishmania major* old world cutaneous leishmaniasis, where no significant difference in efficacy between paromomycin with or without gentamicin was seen.^4^

Currently a phase 3, randomized, double-blind trial to determine if WR 279,396 is superior to paromomycin alone for *L. panamensis* NWCL in Panama is ongoing.^5^ Taking the previous data into account, probably a third therapeutic regimen based on paromomycin 15% plus MBCL 12% should have been included.

Finally, we would like to mention that Sosa and others^1^ assert that their entry criteria of < 10 cutaneous lesions and no evidence of systemic dissemination conferred a very low risk for future mucosal infection. However, other authors consider local therapy for NWCL caused by *L. braziliensis* and *L. panamensis* unsuitable because of the potential risk of metastasis or secondary mucosal spread, and local therapy is recommended only for patients with ≤ 4–5 lesions.^6^,^7^

New clinical trials comparing local treatments for *L. panamensis* NWCL with long follow-up periods to determine the risk of mucosal dissemination are necessary.
